# Core-shell homojunction silicon vertical nanowire tunneling field-effect transistors

**DOI:** 10.1038/srep41142

**Published:** 2017-01-23

**Authors:** Jun-Sik Yoon, Kihyun Kim, Chang-Ki Baek

**Affiliations:** 1 Department of Creative IT Engineering and Future IT Innovation Lab, Pohang University of Science and Technology, Pohang 790-784, Korea; 2 Department of Electrical Engineering, Pohang University of Science and Technology, Pohang 790-784, Korea

## Abstract

We propose three-terminal core-shell (CS) silicon vertical nanowire tunneling field-effect transistors (TFETs), which can be fabricated by conventional CMOS technology. CS TFETs show lower subthreshold swing (*SS*) and higher on-state current than conventional TFETs through their high surface-to-volume ratio, which increases carrier-tunneling region with no additional device area. The on-state current can be enhanced by increasing the nanowire height, decreasing equivalent oxide thickness (EOT) or creating a nanowire array. The off-state current is also manageable for power saving through selective epitaxial growth at the top-side nanowire region. CS TFETs with an EOT of 0.8 nm and an aspect ratio of 20 for the core nanowire region provide the largest drain current ranges with point *SS* values below 60 mV/dec and superior on/off current ratio under all operation voltages of 0.5, 0.7, and 1.0 V. These devices are promising for low-power applications at low fabrication cost and high device density.

14-nm node fin-shaped field-effect transistors (FinFETs) have been introduced by adopting self-aligned double patterning for high integration and air-gapped interconnects to improve AC performance under operation voltage (*V*_DD_) scaling until 0.7 V^1^. Ultra-thin fin width under high aspect ratio is also effective to enhance gate-to-channel controllability and obtain additional DC performance gains. However, thermionic emission transport, which all conventional metal-oxide semiconductor FETs (MOSFETs) follow, has a fundamental limit of 60 mV/dec for subthreshold swing (*SS*) at room temperature; satisfying high drive current while maintaining low leakage current under the *V*_DD_ scaling is certainly difficult.

Meanwhile, tunneling FETs (TFETs) have been considered as one of the promising alternatives to attain *SS* below 60 mV/dec and high on/off current ratio under low *V*_DD_ for mobile applications^2^,^3^,^4^,^5^,^6^,^7^,^8^,^9^,^10^,^11^,^12^,^13^,^14^,^15^. TFETs obey tunneling transport at the source/channel junction by adopting different types of doping between the source and the drain as p-i-n structure. However, small on-state currents (*I*_on_) of the TFETs are still challenging while maintaining low off-state currents (*I*_off_) concurrently. There have been several techniques to improve the *I*_on_ values of the TFETs in the literature. First, the electric field at the tunneling junction was increased through silicided source^5^, pocket between the source and channel^6^ or micro-annealing for abrupt source/channel junction^9^. Second, the tunneling area was increased through adaptation of a vertical nanowire structure to increase the device density and gate-to-channel controllability^4^,^7^ or modulation of the tunneling junction^8^. Additionally, equivalent oxide thickness (EOT) scaling or heterojunction using Ge or III-V materials was also shown to improve the DC performance by increasing gate-to-channel modulation or by decreasing tunneling barriers through low energy bandgap materials and tunneling masses, respectively^13^,^14^,^15^. However, all these lateral TFETs perpendicular to the gate electric field pose challenges to obtain precise the source/channel junction aligned to the gate region for high *I*_on_^16^. Abrupt source/channel junction and highly-doped source region are also required to boost *I*_on_ greatly. In addition, the reliability and variability problems of Ge^17^ and III-V^18^ materials remain unsolved.

Here we propose three-terminal silicon-based homojunction TFETs adopting a core-shell (CS) vertical nanowire structure and compare their DC characteristics to the conventional TFETs for different geometrical parameters such as the nanowire diameter (*D*_NW_) and height (*H*_NW_). CS TFETs are also compared to other silicon-based homojunction TFETs in terms of DC performance metrics such as *I*_on_, *I*_off_, and point *SS*.

## Results

The point *SS* was extracted using two adjacent gate voltages (*V*_gs_) and drain currents (*I*_ds_) as


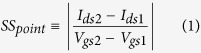


*V*_gs_ was swept from 0 to 1.5 V in steps of 0.01 V, and the drain voltage (*V*_ds_) was tested at 0.5, 0.7, and 1.0 V. To compare the DC performance metrics, conventional TFETs with the *D*_NW_ of 5, 10, 20 nm and *H*_NW_ of 20, 50, 100 nm were considered where superior *I*_ds_ and transconductance were attained^19^; the CS TFETs with *D*_NW_ of 10, 20, 30 nm and *H*_NW_ of 200, 400, 600 nm for the core region were simulated with the same aspect ratio of (*H*_NW_ over *D*_NW_) 20 which is acceptable for the nanowire formation^20^. The shell thickness (*T*_shell_), the epi thickness (*T*_epi_), and the EOT of the CS TFETs were fixed at 5, 20, and 1 nm, respectively, unless specified otherwise. *I*_ds_ values were normalized to the perimeter of the nanowire (*D*_*NW*_ × π) for fair comparison between conventional and CS TFETs.

**Figure 1:**
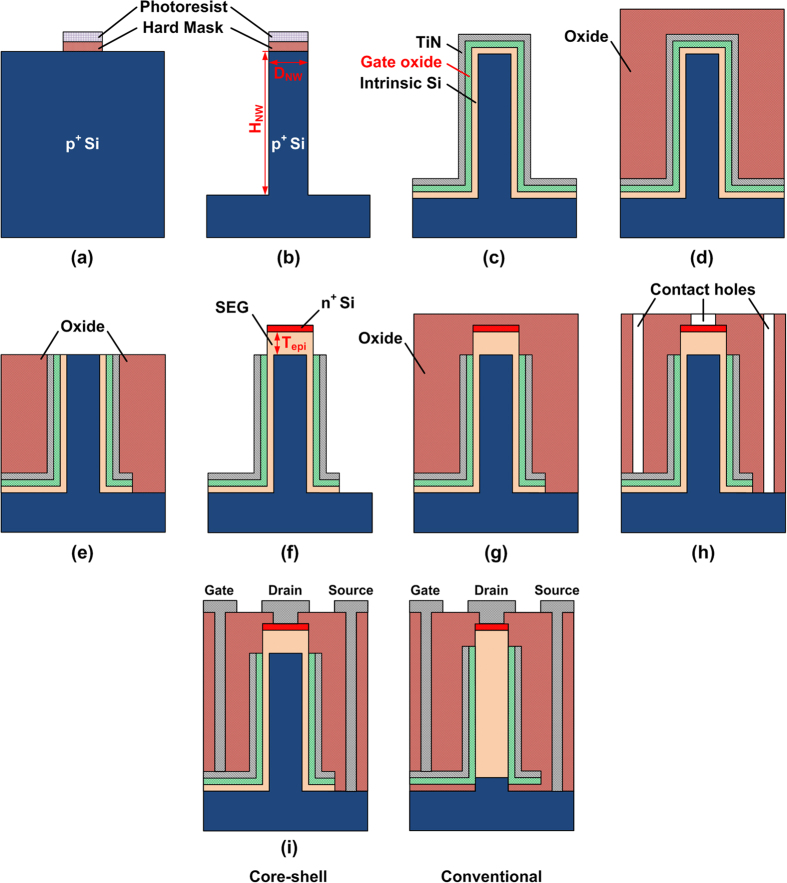
Simple process flow for the core-shell (CS) vertical nanowire TFETs under conventional CMOS technology. (**a**) photoresist and hard-mask defined on highly-doped silicon substrate, (**b**) nanowire formation using anisotropic etching, (**c**) successive deposition of undoped silicon, high-k gate oxide (or rapid thermal oxidation for SiO_2_ gate oxide), and titanium nitride as a metal gate, (**d**) deposition of the oxide material and (**e**) chemical mechanical polishing to expose the silicon regions, (**f**) successive selective epitaxial growth of undoped and highly-doped silicon at the exposed silicon regions only, (**g**) deposition of the oxide material, (**h**) formation of the contact holes, and (**i)** metal contacts. Schematic diagram of the conventional vertical nanowire TFETs is also shown at the bottom. Varying parameters are indicated as red color.

Figure 1 shows the feasibility of CS homojunction silicon vertical nanowire TFETs under conventional CMOS technology. After silicon substrate is highly doped with boron (for n-type TFETs) or with arsenic/phosphorus (for p-type TFETs), hard mask is deposited and then etched to define a vertical nanowire (Fig. 1a). A vertical nanowire with high aspect ratio is formed by inductively coupled plasma reactive ion etching, followed by advanced rinse and dry process such as critical point drying^21^, super-critical drying^22^, or Marangoni drying^23^ to prevent the nanowire collapse (Fig. 1b). Then, low-pressure chemical vapor deposition (LPCVD) or ultra-high vacuum CVD (UHVCVD) is used to deposit undoped poly-crystalline or intrinsic silicon, respectively. Here all the CS TFETs assume to have single-crystal silicon unless specified otherwise. Atomic-layer deposition is done to form high-k gate oxide and titanium nitride metal gate regions successively (Fig. 1c). For SiO_2_ as a gate oxide, rapid thermal oxidation is used instead^24^. The core and shell regions indicate highly-doped and undoped silicon regions, respectively. After etching one-side region for the formation of the source metal contact and depositing oxide material (Fig. 1d), upper parts of the vertical nanowire regions are etched to expose the core and shell silicon regions using chemical mechanical polishing (Fig. 1e). Intrinsic undoped and highly-doped silicon are grown using selective epitaxial growth (SEG) successively for Ohmic contact formation (Fig. 1f). After depositing oxide material (Fig. 1g) and forming contact holes (Fig. 1h), finally, metal contacts are formed for the gate, drain, and source regions (Fig. 1i). Conventional vertical nanowire TFETs are also feasible under slightly different but compatible CMOS process flow^4^,^7^.

**Figure 2:**
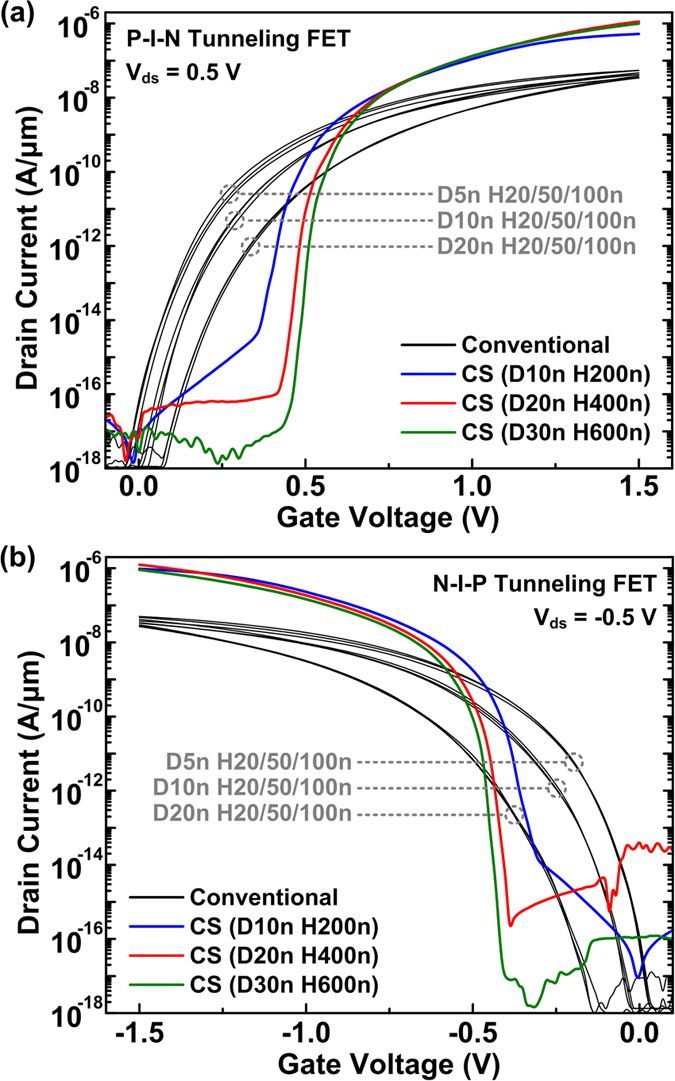
Transfer characteristics of conventional and CS TFETs with different diameters (*D*_NW_) and heights (*H*_NW_) for (**a**) n-type and (**b**) p-type operations.

3-D numerical simulation results show superior transfer characteristics for CS TFETs (colored) compared to conventional TFETs (black) for both n-type (Fig. 2a) and p-type (Fig. 2b) operations at *V*_ds_ of ±0.5 V. The conventional TFETs show lower *I*_ds_ for greater *D*_NW_ because the electric field at the source/channel junction decreases as the gate-to-channel controllability degrades. The transfer characteristics of the conventional TFETs are independent of *H*_NW_ in this sub-μm range^19^ since the *I*_ds_ is dominantly affected by tunneling at the source/channel junction, not by the series resistance at the source, channel, and drain regions unless there is direct tunneling from source to drain in the case of ultra-short *H*_NW_ values^16^.

**Figure 3:**
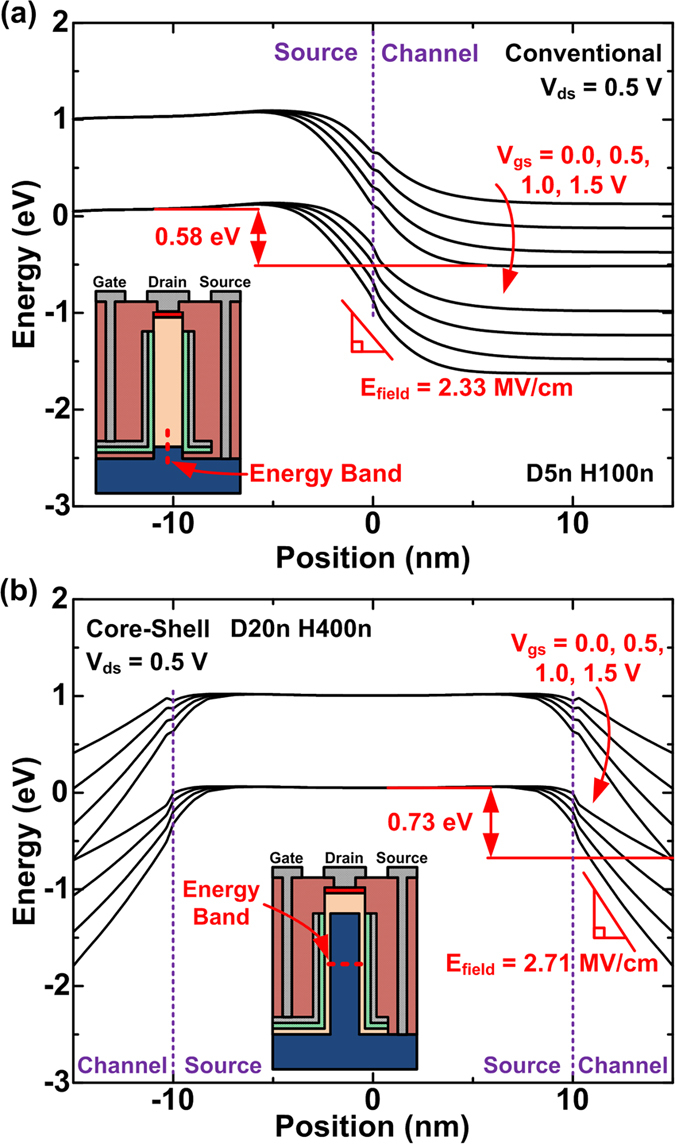
Energy band diagrams at the source/channel junctions for (**a**) conventional and (**b**) CS TFETs at different gate voltages (*V*_gs_) of 0.0, 0.5, 1.0, 1.5 V and the fixed drain voltage (*V*_ds_) of 0.5 V. Inset indicates where the energy band diagrams are from.

Figure 3 shows the energy band diagrams at the source/channel junctions for conventional (*D*_NW_ = 5 nm, *H*_NW_ = 100 nm) and CS (*D*_NW_ = 20 nm, *H*_NW_ = 400 nm) TFETs at *V*_gs_ values of 0.0, 0.5, 1.0, and 1.5 V. The chosen *D*_NW_ and *H*_NW_ values are just one set of examples; other sets of *D*_NW_ and *H*_NW_ show similar band bending phenomena. The energy band diagrams are positioned at where the high band-to-band (BTB) generation rates are obtained. A sudden flexion of the energy bands near the source/channel junctions is seen because the highly-doped source regions have a smaller bandgap affected by the bandgap narrowing model. At a *V*_gs_ of 1.5 V, the CS TFETs show higher electric field of 2.71 MV/cm than the conventional TFETs because the gate electric field is exactly parallel to the electric field at the source/channel junctions^25^,^26^. In addition, more electron-hole pairs can do BTB tunneling (BTBT) for the CS TFETs because of their larger energy difference of 0.73 eV between the conduction and valence bands. In contrast, the CS TFETs have smaller maximum BTB generation rate than the conventional TFETs at a fixed *V*_gs_ because the tunneling carriers in the channel region are slightly apart from the silicon/insulator interface. The charge centroid by the quantum effects reduces the energy differences and thus the amount of the tunneling carriers. Because of the charge centroid, much steeper band bending at the channel region is required to initiate BTB generation for the device operation, so the CS TFETs need a greater turn-on voltage than the conventional TFETs as shown in Fig. 2. Nevertheless, the CS TFETs have a significantly wider source/channel junction for the BTBT at the nanowire sidewalls, thus increasing the *I*_on_ (Fig. S1).

## Discussion

**Figure 4:**
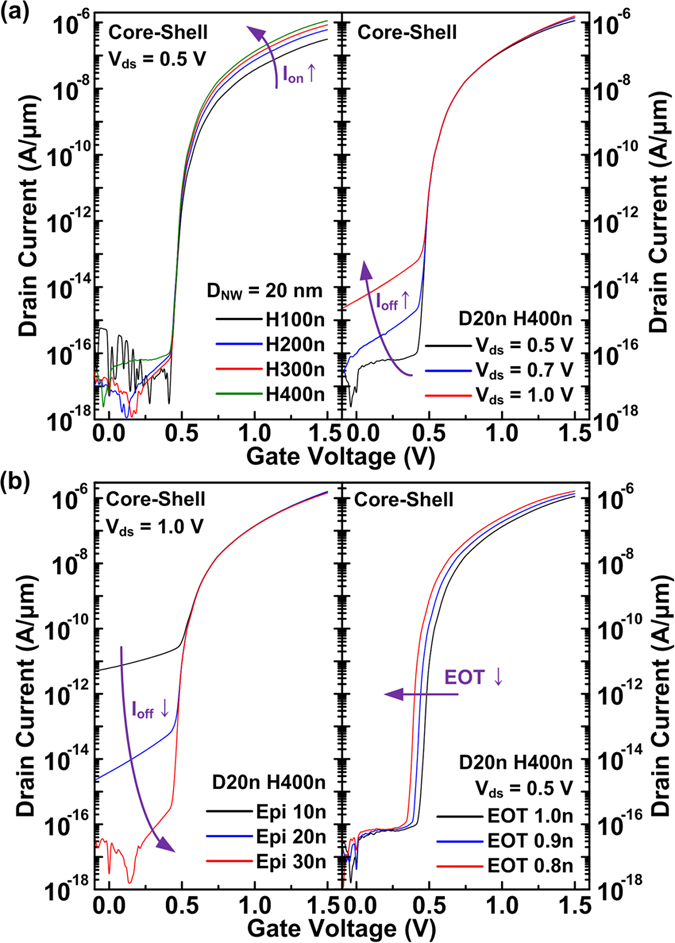
Transfer characteristics of the CS TFETs with (**a**) different *H*_NW_ and *V*_ds_ values and (**b**) different epi thickness (*T*_epi_) and equivalent oxide thickness (EOT).

Detailed investigations of the transfer characteristics for the CS TFETs in terms of the *H*_NW_, *V*_ds_, *T*_epi_, and EOT are shown in Fig. 4. At a fixed *D*_NW_ of 20 nm, the CS TFETs with greater *H*_NW_ show an increased *I*_on_ due to the increased sidewall BTBT regions. Since the *I*_off_ variations are affected by the tunneling currents at the top-side core regions mostly, similar off-state characteristics are obtained for different values of *H*_NW_. Greater *V*_ds_ induces strong band-bending at the top-side core/epi junction and increases *I*_off_ greatly without improving the *I*_on_ much as shown in Fig. 4a. Although increasing *V*_ds_ or *V*_DD_ deviates from the main purpose to reduce the power consumption of the devices, this effect would limit the *I*_on_/*I*_off_ ratio enhancement. But increasing *T*_epi_ from 10 to 30 nm helps to reduce the *I*_off_ critically from 10^−11^ to 10^−16^ A/μm with a slight *I*_on_ degradation as shown in the left of Fig. 4b. The BTB generation rate at the top-side core/epi junction decreases as the *T*_epi_ increases because the band bending at the top-side epi region induced by the high *V*_ds_ is alleviated at the off-state condition (Fig. S2). Lower *SS*_point_, higher *I*_on_, and higher *I*_on_/*I*_off_ ratio are obtained as the EOT decreases by adopting high-k dielectric materials. The EOT is scaled up to 0.8 nm because it is feasible and physically reliable to maintain low gate leakage current of the nanowire structure fabricated by the top-down approach^18^,^27^,^28^. When the gate-to-channel controllability increases for thinner EOT, more abrupt band-bending at the source/channel junction increases the electric field and thus the *I*_on_. Hence, the CS TFETs can achieve superior transfer characteristics when *H*_NW_ increases and the EOT decreases under certain *V*_ds_ and *T*_epi_ values for low *I*_off_.

**Figure 5:**
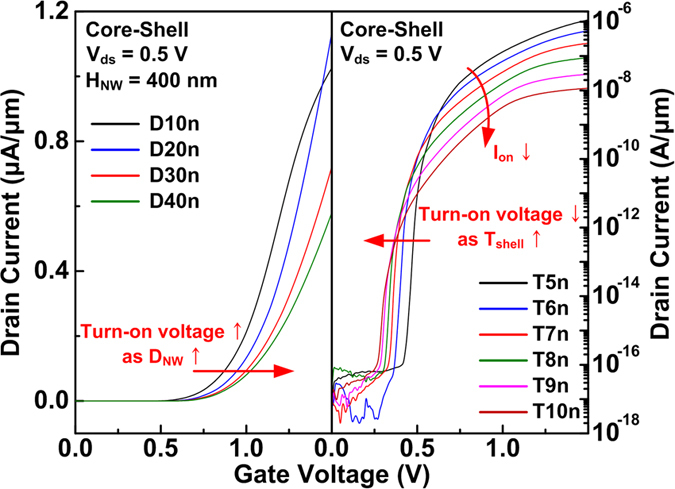
Transfer characteristics of the CS TFETs with different values of *D*_NW_ and shell thickness (*T*_shell_).

The transfer characteristics of the CS TFETs change with *D*_NW_ or *T*_shell_ (Fig. 5). Devices with *D*_NW_ of 10 nm reduce the *I*_ds_ at high *V*_gs_ because of insufficient number of carriers (holes for n-type and electrons for p-type operations) at the core region for the BTBT. The decrease of *I*_on_ for greater *T*_shell_ and the decrease of turn-on voltage to initiate the BTB generation for smaller *D*_NW_ or greater *T*_shell_ can be explained in terms of simple lumped resistance-capacitance (RC) model (Fig. S3) and the energy band diagrams (Fig. S4). Assuming that *V*_ds_ is small and/or *T*_epi_ is thick enough to neglect the capacitive effect by the drain, there are series of resistances and capacitances between the gate and source terminals: the resistance existing at the core region (*R*_core_), and capacitances at the insulator (*C*_in_), depletion at the shell region (*C*_dep_) and the source/channel junction (*C*_tunnel_). The best performance of the CS TFETs can be attained when *V*_gs_ is applied mostly at the *C*_tunnel_ for higher electric field at the source/channel tunnel junction. According to the voltage divider, increasing *C*_in_ and *C*_dep_ and decreasing *C*_tunnel_ and *R*_core_ are desirable to improve the *I*_on_.

In accordance with the simple RC model, the variations of the transfer curves for different geometrical parameters (Figs 4 and 5) can be explained in detail. Decreasing EOT through high-k dielectric materials increases *C*_in_ and improves DC characteristics as shown in Fig. 4b. Lower turn-on voltage for smaller EOT can also be explained by an increase in the voltage at the source/channel junction for the same *V*_gs_ by increasing *C*_in_.

The *R*_core_ from the source/channel junction to the center of the core nanowire increases with *D*_NW_. So, a high *V*_gs_ is required to apply the same electric field at the source/channel junction and initiate the BTB generation as shown in Fig. 5. But the increase of *R*_core_ for greater *D*_NW_ up to 40 nm does not affect the *I*_on_ much because the capacitance is dominant in this *D*_NW_ range. Except for the *D*_NW_, the way to decrease the *R*_core_ is to dope the core nanowire region highly.

As *T*_shell_ increases, the *I*_on_ decreases because the *C*_dep_ decreases and the tunneling length increases, and the tunneling current has an exponential dependence on the tunneling length (Fig. S4). Greater *T*_shell_ induces the BTB generation at lower *V*_gs_ (or turn-on voltage) even though the longer tunneling distance increases the *SS*_point_ at the beginning of the device operation as shown in Fig. 5. Decreasing *T*_shell_ is preferred to increasing the *I*_on_, but too thin *T*_shell_ of several nanometers can increase the energy bandgap due to the quantum confinement effects^29^, which is disadvantageous for BTBT.

Temperature dependence of the performance of conventional and CS TFETs at *V*_ds_ of 0.5 V is investigated in Figs S5 and 6, respectively. For both devices, *I*_off_ increases as temperature increases to 400 K because the enhanced SRH generation increases free carriers inside the channel region^30^,^31^,^32^,^33^,^34^,^35^,^36^. A slight increase of *I*_on_ at elevated temperature is due to the bandgap narrowing effect and thus the decreased energy barrier height at the source/channel junction. Subthreshold characteristics of both devices are independent of temperature because the source/channel junction and Si/SiO_2_ interface are assumed to have no trap density and thus no trap-assisted tunneling (TAT) which is strongly dependent on temperature^31^,^32^,^33^,^34^,^35^. At *V*_ds_ of 0.7 and 1.0 V for the CS TFETs with the *T*_shell_ of 20 nm, the enhanced BTBT at the off state in the top-side intrinsic region screens the SRH generation effect and induces the same temperature dependence as the *I*_on_ (Fig. S6).

CS TFETs having poly-Si shell regions are also investigated to understand how the crystal quality of the shell region affects the DC characteristics (Fig. S7). There are three kinds of grain boundaries (GBs) aligned at different positions within the shell region: (1) the interface between core and shell regions, (2) the middle between core and gate oxide, and (3) the middle half, which are indicated as red dotted line in Fig. S7a. All the cases degrade the DC performance by increasing *I*_off_ and decreasing *I*_on_. Especially, the CS TFETs of the case 2 have the worst *I*_on_/*I*_off_ ratio, about 4 orders lower than the CS TFETs with no GB. At off state, greater number of electrons and holes generated at the GB are accumulated close to the gate oxide and the core region, respectively, by the electric field at the shell region, thus contributing to *I*_off_ greatly. At on state, the BTBT is impeded greatly by the barrier height of the GB. For case 1, the BTBT along with the TAT at the same interface leads to the early onset of device operation. Therefore, it is required to secure the shell region made of high-quality single-crystal silicon to achieve the best DC performance.

**Figure 7:**
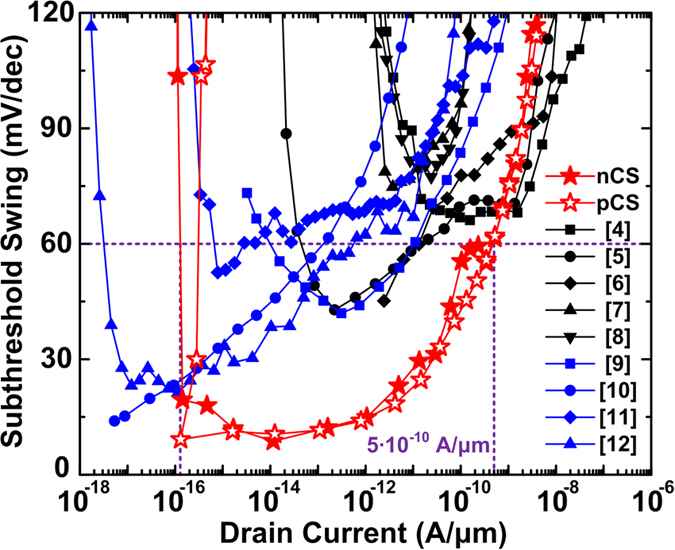
Point subthreshold swing with respect to the drain currents for the best CS TFETs (*D*_NW_ = 20 nm, H_NW_ = 400 nm, T_epi_ = 20 nm, EOT = 0.8 nm) at V_ds_ of 0.5 V (red), the measured data (black) and the simulated data (blue).

Figure 7 shows the *SS*_point_ with respect to the *I*_ds_ of the CS TFETs in this work (red), the measured^4^,^5^,^6^,^7^,^8^ (black) and the simulated^9^,^10^,^11^,^12^ (blue) silicon homojunction TFETs. The CS TFETs in this work have a *D*_NW_ of 20 nm, *H*_NW_ of 400 nm, *T*_shell_ of 5 nm, *T*_epi_ of 20 nm, and EOT of 0.8 nm at *V*_ds_ of 0.5 V. All the measured and simulated *I*_ds_ are normalized with respect to the device width for planar and to the perimeter for nanowire devices. The simulated data^9^,^10^,^11^,^12^ show a sub-60-mV/dec of *SS*_point_ at lower *I*_ds_ ranges, which makes it difficult to substitute the conventional MOSFETs with respect to device operation speed. The measured data^4^,^5^,^6^,^7^,^8^ show better subthreshold characteristics at high *I*_ds_ ranges, but mostly-high *SS*_point_ for the entire *I*_ds_ range is not promising for low-power applications. Among all the *SS*_point_ data, both n-type and p-type CS TFETs show the widest *I*_ds_ range from 10^−16^ to 5·10^−10^ A/μm under an *SS*_point_ of 60 mV/dec which is advantageous for higher *I*_on_ and *I*_on_/*I*_off_ ratio compared to any previously-reported data^4^,^5^,^6^,^7^,^8^,^9^,^10^,^11^,^12^. In addition, both n-type and p-type CS TFETs show comparable transfer characteristics and thus are applicable to the CMOS inverter with one-to-one device ratio.

Table 1 summarizes the *V*_DD_, *I*_on_, and *I*_on_/*I*_off_ ratio for all the data in Fig. 7. The CS TFETs have the same geometrical parameters as in Fig. 7 except for the *T*_epi_ of 30 nm to enhance the off-state characteristics at high *V*_ds_ (Fig. 4b). Both n-type and p-type CS TFETs have comparable *I*_on_ and *I*_on_/*I*_off_, but their lower values are shown in Table 1. After the maximum *I*_on_/*I*_off_ ratios within the *V*_DD_ values are extracted from the transfer curves, the *I*_on_ values at the maximum *I*_on_/*I*_off_ ratios are obtained. All the simulated data^9^,^10^,^11^,^12^ adopt the default values of *A* and *B* for the Kane’s nonlocal BTBT model and thus are comparable to those of the CS TFETs. However, the comparison of DC performance between simulated and measured data is not accurate because the *A* and *B* parameters were not adjusted to the measured data beforehand. But the CS TFETs having an *I*_on_ of 10^−6^ A/μm can achieve high *I*_on_/*I*_off_ ratio of 10^10^ at *V*_ds_ of 1.0 V, which has sufficiently-low *I*_off_ compared to the measured data^4^,^5^,^6^,^7^,^8^. Some of the simulated data^10^,^12^ show *I*_on_/*I*_off_ ratios similar to the CS TFETs, but their low *I*_on_ values even at high *V*_DD_ would decrease operation speed along with significant power consumption. Compared to the CS TFETs, the TFETs with nanotube structure^12^,^14^ also show the possibility to substitute the conventional MOSFETs through high *I*_on_ and *I*_on_/*I*_off_ ratio, but the process complexity and reliability such as two independent gate terminals for the core and shell regions and ultra-thin EOT of 0.5 nm still need to be solved. Overall, the CS TFETs show comparably high *I*_on_ and *I*_on_/*I*_off_ ratio under all *V*_DD_ of 0.5, 0.7, 1.0 V and also show great potential to increase device density through vertical nanowire structure and to adopt the nanowire array easily due to the compatibility to present a three-terminal transistor platform.

## Conclusion

In summary, CS silicon nanowire TFETs show superior DC characteristics along with feasibility under conventional CMOS technology. They are capable of high *I*_on_, *I*_on_/*I*_off_ ratio and sub-60-mV/dec of *SS*_point_ over 6 orders of *I*_ds_ due to their broad BTBT regions at the nanowire sidewall source/channel junctions. Greater *T*_epi_ through SEG process shows possibilities to reduce the *I*_off_ by preventing band modulation of the top-side channel region induced by the drain. The *I*_on_ and *SS*_point_ are also improved greatly when the height of the core nanowire region increases and the EOT decreases through the use of high-k gate oxide for better gate-to-channel controllability. Decreasing *T*_shell_ also improves the DC characteristics by decreasing tunneling length and *C*_dep_ at the shell region, but it remains a concern for degrading the *I*_on_ because a too thin *T*_shell_ can induce bandgap widening for the shell region due to the quantum confinement effects. Comparing all the previously-reported measured and simulated silicon homojunction TFETs, simple three-terminal CS TFETs achieve the best *I*_on_ and *I*_on_/*I*_off_ ratio under ultra-low *V*_DD_ and also show the accessibility as a nanowire array for the drive current enhancement, thus showing potential for low-power applications.

## Methods

Both conventional and CS nanowire TFETs were simulated using 3-D Sentaurus TCAD^37^ with Kane’s nonlocal band-to-band tunneling (BTBT) model with default parameters (*A* = 4 × 10^14^ cm^−3^·s^−1^, *B* = 1.9 × 10^7^ V·cm^−1^) for silicon as an indirect bandgap material for phonon-assisted tunneling^38^,^39^. Kane’s nonlocal BTBT model is given by


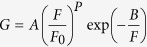


where *G* is BTB generation rate, *F*_0_ = 1 V/cm, *P* = 2.5 for phonon-assisted tunneling, *A* and *B* are Kane parameters, and *F* is the electric field in the tunneling direction. Mobility was calculated using Masetti and Lombardi models for doping-dependence and degradation at the silicon/oxide interface, respectively. Bandgap narrowing model and Fermi-Dirac distribution were also included to calculate the tunneling currents correctly for the degenerate silicon regions. Shockley-Read-Hall (SRH) with doping-dependent lifetime and Auger recombination models were considered as well. In addition, a modified local-density approximation model with six-band k·p for holes and two-band k·p for electrons was used to consider the quantum confinement effects at the nanowire regions along with orientation dependency on the (001) wafer. All these mobility and bandgap-related models include the temperature-dependent factors such as carrier mobility, intrinsic carrier density, SRH generation, and energy bandgap. For the study on poly-crystalline silicon (poly-Si) shell regions, Hurkx trap-assisted tunneling (TAT) model was also included to consider trap density at the grain boundary (GB). It was assumed that there is only a single GB in the shell region, and the trap density of the GB is 10^13^ cm^−2^eV^−1^ within the silicon energy bandgap^40^.

All the simulated n-type and p-type TFETs have the same doping concentrations for the core (source) regions of 10^20^ cm^−3^, for the shell (channel) regions of 10^15^ cm^−3^, and for the drain side of 10^19^ cm^−3^, but they have different types of dopants: the n-type TFETs have p^++^-i-n^+^ structure, whereas the p-type TFETs have n^++^-i-p^+^ structure for the source-channel-drain regions. The drain regions have lower doping concentration than the source to lessen the ambipolar effects at the off-state condition. All the silicon regions are assumed to have uniform and abrupt doping profile. The metal gate work-functions are fixed to 4.2 eV and 5.1 eV for n-type and p-type TFETs, respectively.

## Additional Information

**How to cite this article**: Yoon, J.-S. *et al*. Core-shell homojunction silicon vertical nanowire tunneling field-effect transistors. *Sci. Rep.* **7**, 41142; doi: 10.1038/srep41142 (2017).

**Publisher's note:** Springer Nature remains neutral with regard to jurisdictional claims in published maps and institutional affiliations.

## Supplementary Material

Supplementary Information

## Figures and Tables

**Figure 6:**
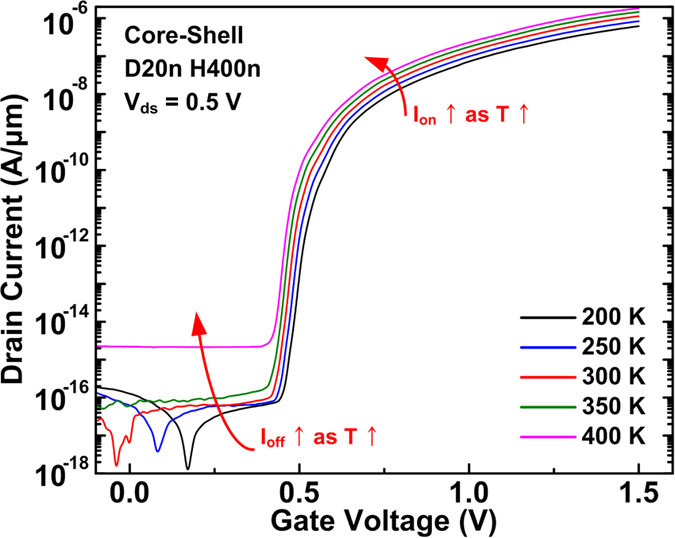
Transfer characteristics of the CS TFETs at the fixed *V*_ds_ of 0.5 V at different temperatures. The devices with different *D*_NW_ and *H*_NW_ show similar temperature dependence and thus are not shown here.

**Table 1:** DC performance metrics of all the silicon homojunction TFETs with different EOT including measured, simulated, and this work.

References	Technology	EOT (nm)	|*V*_DD_| (V)	*I*_on_ (A/μm)	*I*_on_/*I*_off_
4	Vertical NW^a^	4.5	1.2	6 × 10^−6^	4 × 10^6^
5	Planar	0.9	1.0	10^−6^	7 × 10^7^
6	Planar	3.5	1.1	2 × 10^−6^	6 × 10^5^
7	Vertical NW	4.5	0.6	6 × 10^−9^	2 × 10^3^
8	Planar	2.0	0.6	10^−8^	10^4^
9	Horizontal NW	1.3	0.5	2 × 10^−8^	6 × 10^6^
10	Planar	2.0	1.0	10^−8^	3 × 10^9^
11	Planar	~0.5	0.3	10^−9^	2 × 10^3^
12	Vertical NW (NT^b^)	0.5	1.0	3 × 10^−8^	2 × 10^10^
This work	Vertical NW (CS^c^)	0.8	0.5	10^−7^	4 × 10^10^
			0.7	3 × 10^−7^	2 × 10^11^
			1.0	5 × 10^−7^	10^12^

^a^NW is nanowire.

^b^NT is nano-tube structure.

^c^CS is core-shell structure.
