# Supplementary material for: Core-shell homojunction silicon vertical nanowire tunneling field-effect transistors
Source: Sci Rep. 2017 Jan 23;7:41142. doi: 10.1038/srep41142 (PMC5255564; doi:10.1038/srep41142)
Supplement: Supplementary Information [file srep41142-s1.pdf]

Core-shell homojunction silicon vertical nanowire tunneling field-effect transistors

Jun-Sik Yoon[1], Kihyun Kim[1], and Chang-Ki Baek[1, 2]

[1]*Department of Creative IT Engineering and Future IT Innovation Lab, Pohang University of Science and Technology, Pohang 790-784, Korea*

[2]*Department of Electrical Engineering, Pohang University of Science and Technology, Pohang 790-784, Korea*

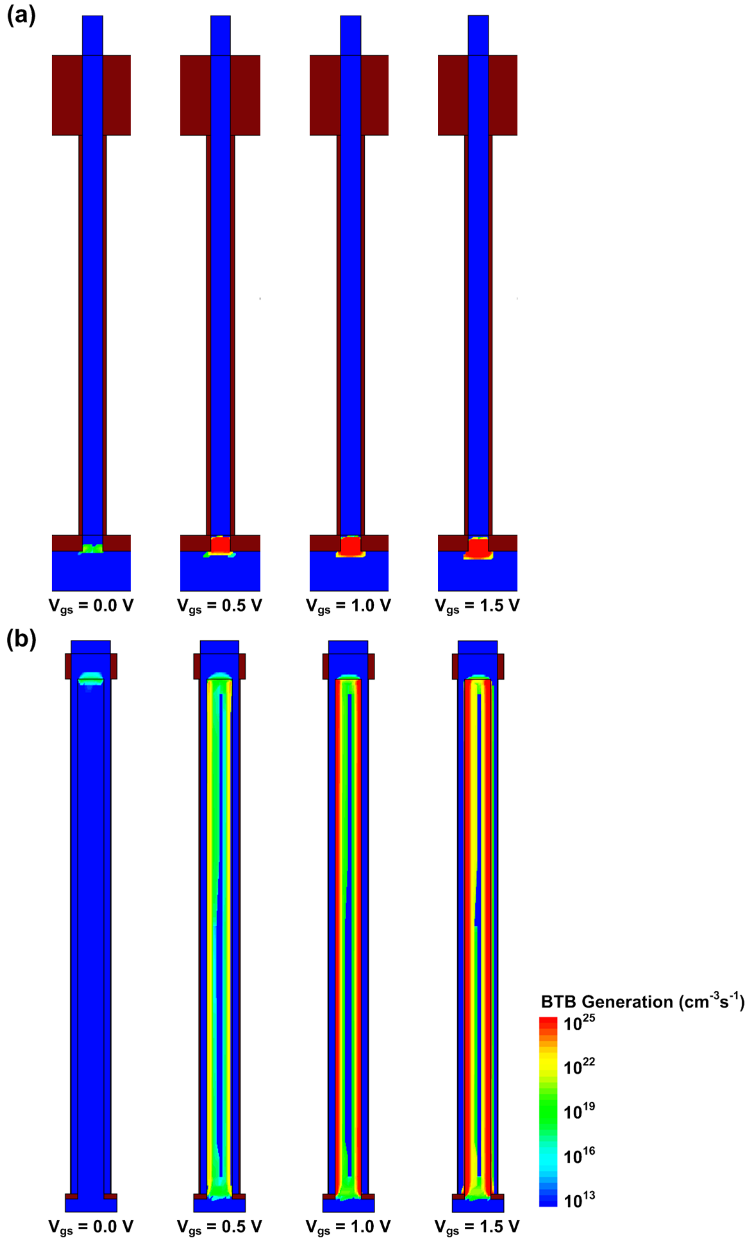

Figure S1. BTB generation rates of the (a) conventional (D_{NW} = 5 nm, H_{NW} = 100 nm) and (b) CS (D_{NW} = 20 nm, H_{NW} = 400 nm) TFETs with different V_{gs} of 0.0, 0.5, 1.0, and 1.5 V at the fixed V_{ds} of 0.5 V

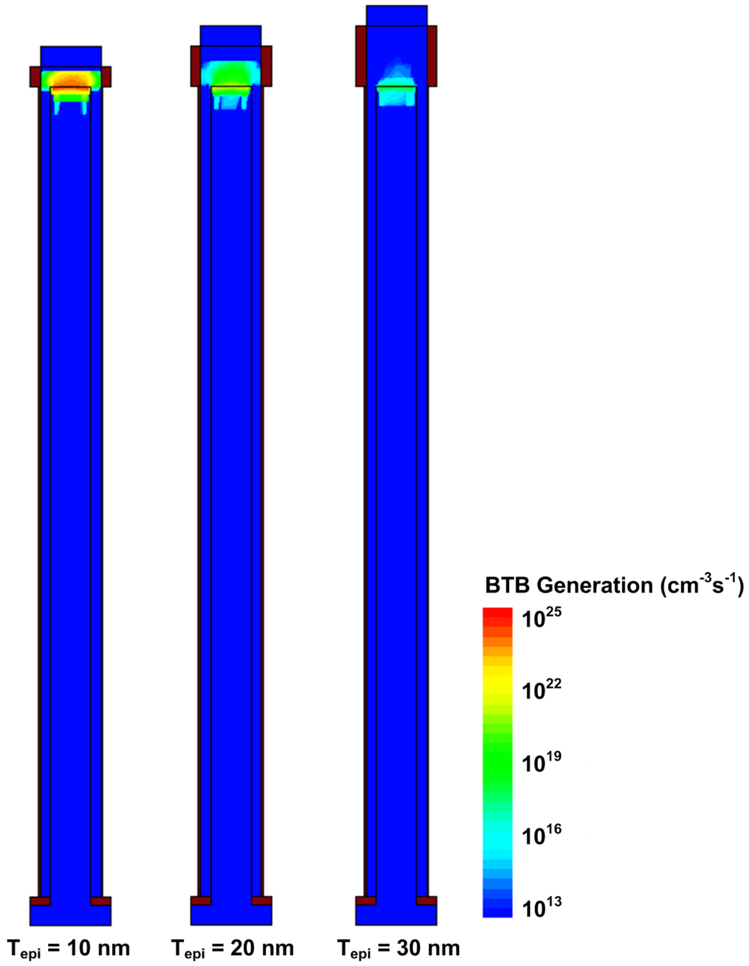

Figure S2. BTB generation rates of the CS (D_{NW} = 20 nm, H_{NW} = 400 nm) TFETs with different epi thickness (T_{epi}) of 10, 20, and 30 nm at off-state condition (V_{gs} = 0.0 V, V_{ds} = 1.0 V)

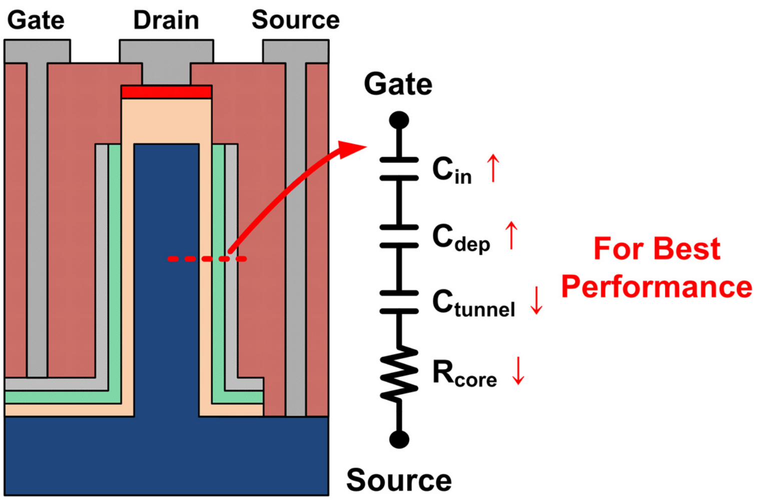

Figure S3. Simple lumped resistance-capacitance model between gate and source terminals. Best performance can be expected when C_{in} and C_{dep} increase and when C_{jn} and R_{core} decrease.

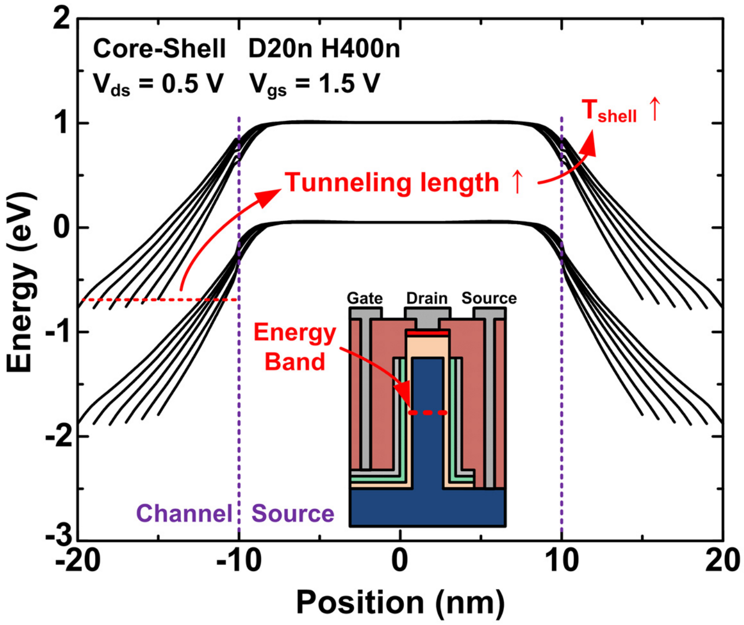

Figure S4. Energy band diagram of the CS (D_{NW} = 20 nm, H_{NW} = 400 nm) TFETs with different T_{shell} of 5, 6, 7, 8, 9, 10 nm at V_{gs} of 1.5 V and V_{ds} of 1.0 V. Tunneling length increases as the T_{shell} increases.

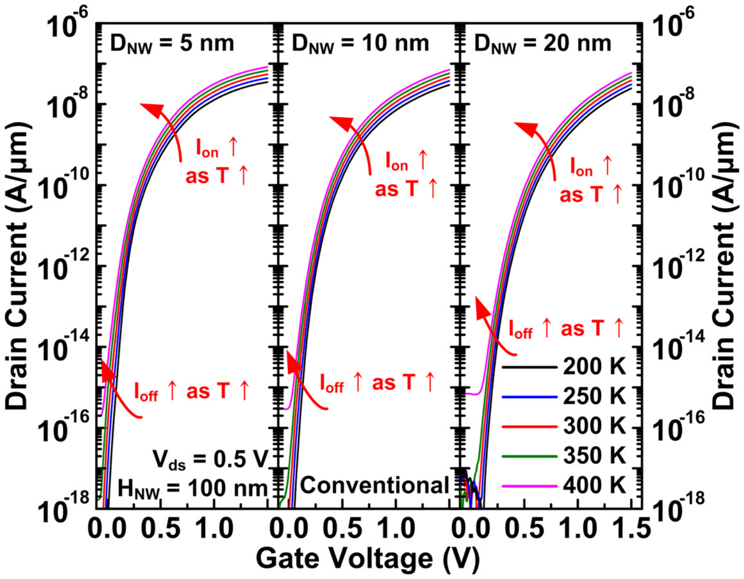

Figure S5. Transfer characteristics of the conventional TFETs with different D_{NW} at V_{ds} of 0.5 V at different temperatures. The devices with different H_{NW} show similar temperature dependence and thus are not shown here.

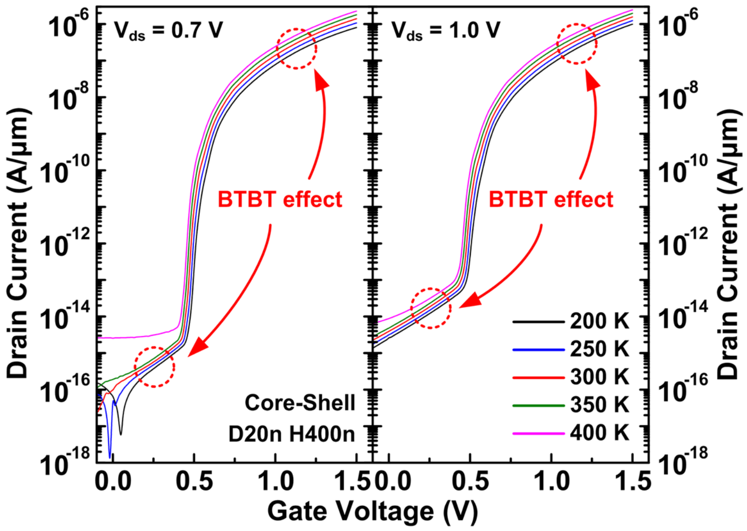

Figure S6. Transfer characteristics of the CS TFETs at V_{ds} of 0.7 and 1.0 V at different temperatures. Both off-state and on-state conditions follow BTBT mechanism and show the same temperature dependence.

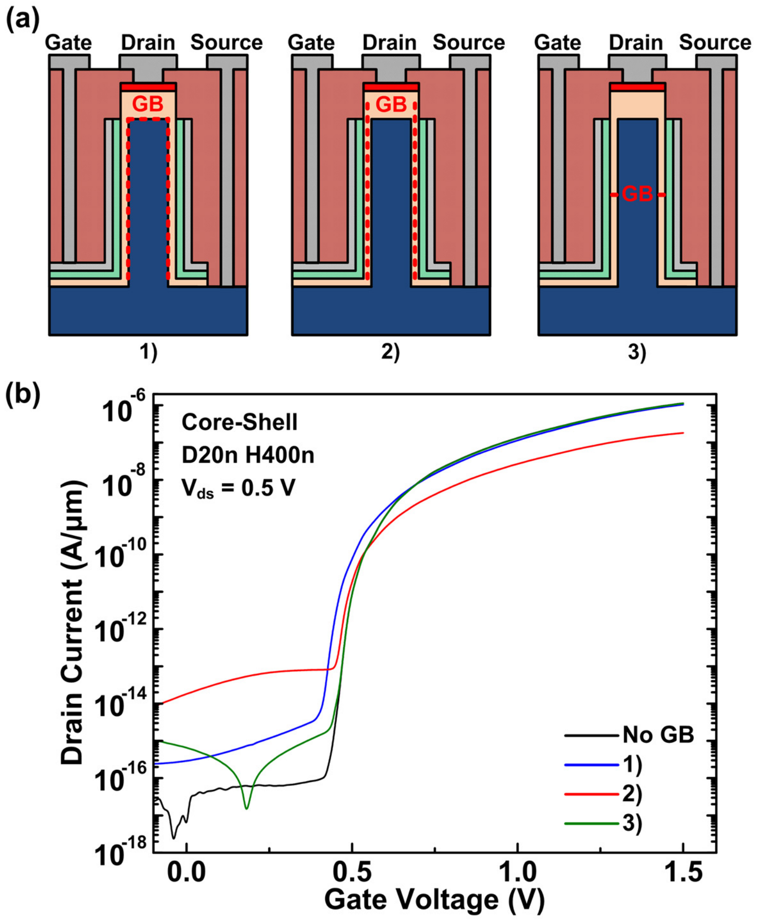

Figure S7. (a) Schematic diagrams of the CS TFETs having poly-Si shell regions with a single GB (specified as red dotted line) aligned at different positions and (b) Transfer characteristics of the CS TFETs at V_{ds} of 0.5 V
